# Looking beyond year 1 in the molecular era of pediatric brain tumor diagnosis: confirmatory testing of germline variants found on tumor sequencing

**DOI:** 10.3389/fonc.2024.1338022

**Published:** 2024-03-06

**Authors:** Brittany L. Greene, Shannon M. Stasi, Michelle A. Ting, Natalie Waligorski, Bonnie L. Cole, Christina M. Lockwood, Vera A. Paulson, Jillian G. Buchan, Amy Lee, Jeffrey G. Ojemann, Richard G. Ellenbogen, Jeffrey Stevens, Sarah E. S. Leary

**Affiliations:** ^1^ Ben Towne Center for Childhood Cancer Research, Seattle Children’s Research Institute, Seattle, WA, United States; ^2^ Department of Pediatrics, University of Washington, Seattle, WA, United States; ^3^ Cancer and Blood Disorders Center, Seattle Children’s Hospital, Seattle, WA, United States; ^4^ Department of Laboratories, Seattle Children’s Hospital, Seattle, WA, United States; ^5^ Department of Laboratory Medicine and Pathology, University of Washington, Seattle, WA, United States; ^6^ Department of Pediatric Neurosurgery, Seattle Children’s Hospital, Seattle, WA, United States; ^7^ Department of Neurological Surgery, University of Washington, Seattle, WA, United States

**Keywords:** childhood cancer, brain tumor, molecular testing, next-generation sequencing, germline

## Abstract

**Purpose:**

Somatic molecular profiling of pediatric brain tumors aids with the diagnosis and treatment of patients with a variety of high- and low-grade central nervous system neoplasms. Here, we report follow-up targeted germline evaluation for patients with possible germline variants following tumor only testing in the initial year in which somatic molecular testing was implemented at a single institution.

**Patients and Methods:**

Somatic testing was completed for all tumors of the central nervous system (CNS) undergoing diagnostic workup at Seattle Children’s Hospital during the study period of November 2015 to November 2016. Sequencing was performed in a College of American Pathologists-accredited, Clinical Laboratory Improvements Amendments-certified laboratory using UW-OncoPlex™ assay (version 5), a DNA-based targeted next generation sequencing panel validated to detect genetic alterations in 262 cancer-related genes. We tracked subsequent clinical evaluation and testing on a subgroup of this cohort found to have potential germline variants of interest.

**Results:**

Molecular sequencing of 88 patients’ tumors identified 31 patients with variants that warranted consideration of germline testing. To date, 19 (61%) patients have been tested. Testing confirmed germline variants for ten patients (31% of those identified for testing), one with two germline variants (*NF1* and mosaic *TP53*). Eight (26%) patients died before germline testing was sent. One patient (13%) has not yet had testing.

**Conclusion:**

Clinically validated molecular profiling of pediatric brain tumors identifies patients who warrant further germline evaluation. Despite this, only a subset of these patients underwent the indicated confirmatory sequencing. Further work is needed to identify barriers and facilitators to this testing, including the role of genetic counseling and consideration of upfront paired somatic-germline testing.

## Introduction

Over the prior two decades, large pediatric oncology sequencing studies have demonstrated the feasibility of upfront genomic testing at the time of high-risk diagnosis and relapse of childhood cancer (1, 2). While access to sequencing via research protocols has increased for children with cancer over the prior decade, there is currently no consensus regarding best-practice diagnostics for routine clinical practice.

NGS assays designed to detect single-nucleotide variants, insertions and deletions, copy number changes, and rearrangements in genes selected for their clinical significance in cancer, can identify alterations that clarify diagnosis or suggest molecularly targeted therapeutics. In some cases, somatic sequencing can also result in secondary findings, identifying variants in the tumor sample that appear likely to be present in the patient’s germline, which may or may not be related to the development of the patient’s cancer (3–5).

Our group previously reported the findings from a prospective cohort study where clinically validated molecular profiling of pediatric brain tumors aided in the diagnosis and treatment of patients with a variety of high- and low-grade primary, newly-diagnosed and relapsed brain tumors (6). A subset of the patients undergoing tumor only testing during the first year in which somatic clinical targeted sequencing was performed were found to have possible germline variants (6). We sought to evaluate how these results may have impacted further clinical evaluation and testing in this subgroup of the cohort.

## Materials and methods

All patients with tumors of the central nervous system (CNS) undergoing diagnostic evaluation at Seattle Children’s Hospital during the study period from November 2015 to November 2016 had their tumors sequenced using the UW-OncoPlex™ assay (version 5; https://testguide.labmed.uw.edu/view/OPX)? (7). This multiplexed targeted next-generation sequencing panel, designed for the detection of genetic alterations in 262 cancer-related genes, is performed in a College of American Pathologists (CAP)-accredited and Clinical Laboratory Improvements Amendments (CLIA)-certified laboratory. Patients with tumor diagnosis by imaging alone were excluded from this study. The patients in this study consented to tumor banking, biology, and return of results (Seattle Children’s Hospital Institutional Review Board No. 00000506). Data were extracted from the medical record.

The molecular tumor results were discussed at a monthly molecular brain tumor conference, including pediatric neuro-oncology, pediatric pathology, molecular pathology, genetic counseling, and neurosurgery along with a research coordinator (6). Each month, all new patients were systematically reviewed in a presentation format consisting of clinical features and magnetic resonance imaging presented by a neuro-oncologist (S.E.S.L.), histologic findings and photomicrographs by a pediatric pathologist (B.L.C.), molecular results by a molecular pathologist (C.M.L.) followed by group discussion including laboratory genetic counseling (S.M.S.). Variants were identified for potential follow-up constitutional testing if they met any of the following criteria: pathogenic or likely pathogenic variants in genes known to be associated with cancer predisposition; or large indels, structural rearrangements or exonic deletions/duplications in genes known to be associated with cancer predisposition; or variants in a patient with clinical features, family history or tumor type suggestive of a constitutional predisposition related to the involved variant (8). The variant allele frequency (VAF) was not used to exclude variants warranting additional follow-up constitutional testing based on other features. Details of the laboratory workflow and methods have previously been published in a Data Supplement (6).

We tracked subsequent clinical evaluation and testing on a subgroup of this cohort who were found to have variants considered to be potentially germline. Individuals in this cohort (or their parents/guardians) were informed of the results and the recommendation for germline testing by their clinical team. Germline confirmatory testing involved the collection a peripheral blood sample or otherwise available non-tumor tissue. Results available in the electronic medical record were tracked on this subgroup of the cohort.

## Results

**Figure 1:**
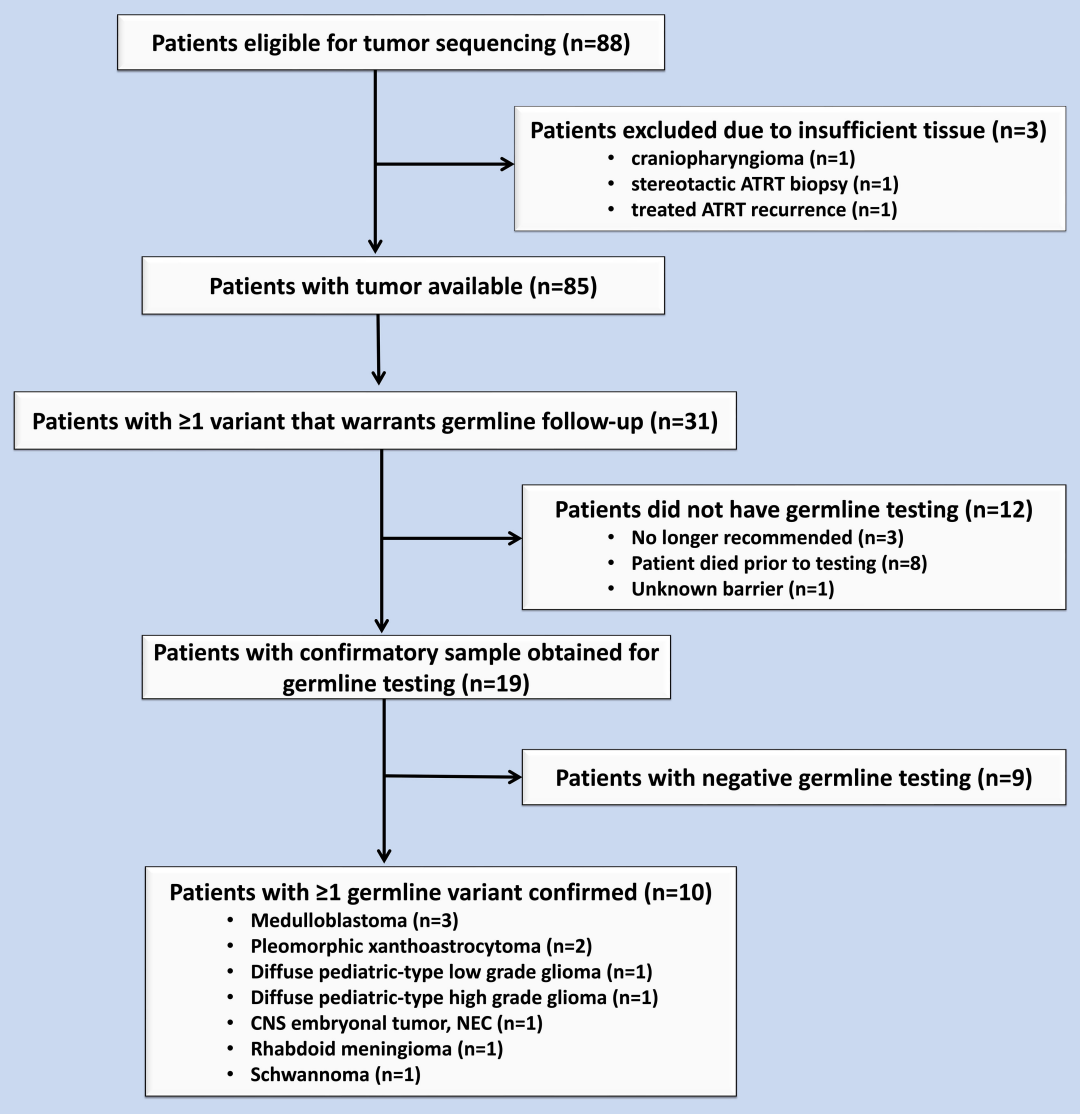
Flow chart of eligible patients during the study period from November 2015 to November 2016.

Molecular sequencing was performed on CNS tumors of 88 patients. At the time of biopsy or surgical resection of their tumor, study participants’ ages ranged from 1 month to 21 years (median 7 years). Sequencing identified 31 patients (35%) whose tumors harbored variants warranting consideration of germline testing (**Figure 1**). The details of these results were previously published (6). To date, 19 patients (61% of those with recommended germline follow-up) have been tested (**Table 1**) (6). Thirteen (68%) of these patients who underwent testing met with an oncology genetic counselor who coordinated testing, while the other patients had the test coordinated by an oncologist at the institution (2, 11%), a provider at another institution (3, 16%), or an established genetic counselor given known germline genetic diagnosis (1, 5%). Germline testing was performed on peripheral blood for 18 patients and on non-affected brain tissue for one patient (patient 7) who had died prior to confirmatory testing. Confirmatory germline testing was performed a median of 13 months following initial tumor testing and ranged from 3 to 57 months. The patients’ ages at the time of confirmatory testing ranged from 9 months to 21 years (median 13 years). Of these 19 patients, testing confirmed germline variants for 10 patients (52% of those tested; 11.4% of the study population) as of the last follow-up. The confirmed germline findings were identified in *ATM*, *BAP1*, *BRIP1*, *FANCA*, *MSH2*, *NF1*, *NF2*, *RAD51D*, *and TP53.* One patient was found to have two germline variants (*NF1* and mosaic *TP53*) (**Table 1**). Two other patients each had two somatic variants warranting testing, with one of the two variants in each patient confirmed to be germline. Of these 11 confirmed germline variants (in 10 patients), three (27%) had a corresponding tumor VAF less than 40%. Of the 10 patients with a confirmed germline variant, 8 patients (80%) received a family referral for genetic counseling and 3 (30%) had at least one relative undergo testing (**Table 1**).

**Table 1:** Description of variants found in tumors prompting recommendation for germline testing.

Patient ID	Tumor	Gene	Variant (p.;c.)	VAF	Germline Variant Classification	Germline Testing Status (reason)	Time from Somatic to Germline Testing (months)	Germline testing*	Cascade Testing Status (reason)
27	Pleomorphic xanthoastrocytoma, CNS WHO Grade 3	*NF1*	p.S812Lfs*8; NM_000267.3:c.2439_2452dup	25%	Pathogenic	Sent	4	Positive – confirmed existing clinical dx	N/A (likely *de novo*)
		*TP53*	p.R110_F113del; NM_000546.5:c.328_339del	23%	Likely Pathogenic	Sent	4	Positive – mosaic	N/A (mosaic)
39	Pleomorphic xanthoastrocytoma, CNS WHO Grade 2	*TP53*	p.R181S; NM_000546.5:c.541C>A	64%	Variant of uncertain significance	Sent	3	Positive	one parent w/cancer negative; siblings and other parent did not pursue testing (unknown)
33	Diffuse pediatric-type high-grade glioma, H3-wildtype and IDH wildtype, CNS WHO grade 4	*MSH2*	deletion exons1-6, NM_000251.2	–	Pathogenic	Sent	17	Positive	one parent negative; other parent did not pursue testing (out of state); sibling referred with testing status unknown (out of state)
		*TP53*	p.R248W; NM_000546.5:c.742C>T	74%	Pathogenic	Sent	17	Negative	–
		*ATM*	p.K2811Sfs*46; NM_000051.3:c.8432del	47%	Pathogenic	Sent	17	Negative	–
57	Medulloblastoma, non-WNT/non-SHH, CNS WHO grade 4	*RAD51D*	p.S111*; NM_002878.3:c.330dup	45%	Pathogenic	Sent	10	Positive	family enrolled in *RAD51D* study; testing status unknown
		*PMS2*	p.K666Rfs*4; NM_000535.5:c.1997_1998del	74%	Likely pathogenic	Sent	10	Negative	–
30	Medulloblastoma, non-WNT/non-SHH, WHO grade IV	*FANCA*	p.R951W; NM_000135.2:c.2851C>T	99%	Pathogenic	Sent	57	Positive	parents referred to genetics; testing status unknown
61	Medulloblastoma, WNT-activated, CNS WHO grade 4	*ATM*	p.F2799Kfs*4; NM_000051.3:c.8395_8404del	25%	Pathogenic	Sent	14	Positive	parents declined testing (focus on immediate clinical concerns in child)
83	CNS Embryonal tumor, NEC	*SUFU*	p.V148M; NM_016169.3:c.442G>A,	52%	Variant of uncertain significance	Sent	6	Positive	one parent positive; grandparent (affected parent’s side) w/cancer negative
66	Rhabdoid meningioma, CNS WHO grade 3	*BAP1*	p.Q392*; NM_004656.3:c.1174C>T	81%	Pathogenic	Sent	4	Positive	referral status unknown (germline testing coordinated by outside provider)
67	Schwannoma, CNS WHO grade 1	*NF2*	p.Q319*; NM_000268.3:c.955C>T	90%	Likely pathogenic	Sent	n/a (germline testing prior to somatic testing)	Positive	family members referred to genetics but not seen (unknown)
74	Diffuse pediatric-type low-grade glioma, MAPK pathway- altered	*BRIP1*	p.R798*; NM_032043.2:c.2392C>T	48%	Pathogenic	Sent	42	Positive	one parent positive; sibling diagnosed with Hodgkin lymphoma is negative
7	Diffuse midline glioma, H3 K27-altered, CNS WHO grade 4	*TP53*	p.C277*; NM_000546.5:c.831T>A	69%	Pathogenic	Sent	18	Negative (unaffected brain tissue)	–
35	Diffuse pediatric-type high-grade glioma, H3-wildtype and IDH wildtype, CNS WHO grade 4	*TP53*	p.V173L; NM_000546.5:c.516_517delinsGT	86%	Likely Pathogenic	Sent	12	Negative	–
75	Choroid Plexus Carcinoma, CNS WHO grade 3	*TP53*	p.E224V; NM_000546.5:c.671A>T	96%	Variant of uncertain significance	Sent	3	Negative	
41	Adult-type diffuse glioma, IDH-mutant, CNS WHO grade 2	*TP53*	p.R273H; NM_000546.5:c.818G>A	64%	Pathogenic Variant of uncertain significance	Sent	31	Negative	–
		*SMARCB1*	p.V136L; NM_001007468.1:c.406G>T	42%		Not sent (no longer indicated)	–	n/a	–
29	Atypical teratoid/rhabdoid tumor, CNS WHO grade 4	*SMARCB1*	p.P374Rfs*100; NM_001007468.1:c.1121delC	59%	Pathogenic	Sent	5	Negative	–
47	Medulloblastoma, SHH-activated and TP53-wildtype, CNS WHO grade 4	*PTCH1*	p.K838Sfs*64; NM_000264.3:c.2513_2516del	77%	Likely Pathogenic	Sent	32	Negative	–
69	Medulloblastoma, SHH-activated and *TP53*-mutant, CNS WHO grade 4	*PTCH1*	p.L90Nfs*28; NM_000264.3:c.267_268insAA	84%	Likely Pathogenic	Sent	7	Negative	–
		*TP53*	p.V274A; NM_000546.5:c.821T>C	87%	Pathogenic	Sent	7	Negative	–
81	Medulloblastoma, SHH-activated and *TP53*-wildtype, CNS WHO grade 4	*SUFU*	NM_016169.3:Deletion	–	Pathogenic	Sent	22	Negative	–
73	Spinal Ependymoma, CNS WHO grade 2	*NF2*	p.A193Vfs*11; NM_000268.3:c.574_577dup	71%	Likely Pathogenic	Sent	29	Negative	–
76	Medulloblastoma, non-WNT/non-SHH, CNS WHO grade 4	*RAD51C*	p.I144T; NM_058216.1:c.431T>C	70%	Likely Benign	Not sent (unknown)	–	–	–
48	Desmoplastic Infantile Ganglioglioma/Astrocytoma, CNS WHO grade 1	*FGFR3*	p.A374G; NM_000142.4:c.1121C>G	54%	Likely Benign	Not sent (no longer indicated)	–	–	–
15	Pilocytic astrocytoma, CNS WHO grade 1	*DYPD*	NM_000548.3:c.4959C>T, IVS14 + 1 G>A	51%	Pathogenic	Not sent (no longer indicated)	–	–	–
53	Medulloblastoma, non-WNT/non-SHH, CNS WHO grade 4	*FBXW7*	p.G423V; NM_033632.3:c.1268G>T	47%	Variant of uncertain significance Variant of uncertain significance Likely Pathogenic	Not sent (no longer indicated)	–	–	–
		*FBXW7*	p.R689Q; NM_033632.3:c.2066G>A	41%		Not sent (no longer indicated)	–	–	–
		*STAG2*	p.R967*; NM_006603.4:c.2899A>T	97%		Not sent (no longer indicated)	–	–	–
21	Diffuse midline glioma, H3 K27-altered, CNS WHO grade 4	*TP53*	p.C277F; NM_000546.5:c.830G>T	52%	Likely Pathogenic	Not sent (patient died)	–	–	–
63	Medulloblastoma, large cell/anaplastic, SHH-activated and *TP53*-mutant, CNS WHO grade 4	*SUFU*	p.L371Q; NM_016169.3:c.1112T>A	65%	Variant of uncertain significance	Not sent (patient died)	–	–	–
		*TP53*	p.R342*; NM_000546.5:c.1024C>T	13%	Pathogenic	Not sent (patient died)	–	–	–
85	Diffuse midline glioma, H3 K27-altered, CNS WHO grade 4	*TP53*	p.P128Lfs*42; NM_000546.5:c.383del	47%	Pathogenic	Not sent (patient died)	–	–	–
77	Diffuse midline glioma, H3 K27-altered, CNS WHO grade 4	*TP53*	p.G266R; NM_000546.5:c.796G>A	70%	Likely Pathogenic	Not sent (patient died)	–	–	–
68	Diffuse pediatric-type high-grade glioma, H3-wildtype and IDH-wildtype, CNS WHO grade 4	*NF1*	p.R1947*; NM_000267.3:c.5839C>T	78%	Pathogenic	Not sent (patient died)	–	–	–
		*TP53*	p.R158G; NM_000546.5:c.472C>G	73%	Pathogenic	Not sent (patient died)	–	–	–
12	Medulloblastoma, SHH-activated and *TP53*-wildtype, CNS WHO grade 4	*PTCH1*	p.V908Gfs*8; NM_000264.3:c.2720dupT	13%	Likely Pathogenic	Not sent (patient died)	–	–	–
78	Medulloblastoma, SHH-activated and *TP53*-mutant, CNS WHO grade 4	*TP53*	p.R175H; NM_000546.5:c.524G>A	86%	Pathogenic	Not sent (patient died)	–	–	–
		*SUFU*	NM_016169.3:Deletion	–	Pathogenic	Not sent (patient died)	–	–	–
3	Pilocytic astrocytoma, CNS WHO grade 1	*NF1*	p.N2364Kfs*12; NM_000267.3:c.7089_7090insTT	63%	Pathogenic	Not sent (patient died)	–	–	–

CNS, central nervous system; VAF, variant allele frequency; dx, diagnosis; n/a, not applicable.

*Confirmatory germline testing was performed on peripheral blood samples, unless otherwise noted.

For some patients, confirmation of a germline variant clarified their diagnosis or was relevant to their treatment plan. Sequencing of one patient’s high grade pleomorphic xanthoastrocytoma (PXA) demonstrated a somatic *BRAF* variant and a *TP53* variant, which was confirmed to be present in the germline (patient 39, **Table 1**). These findings directly impacted their treatment plan, with the use of a *BRAF* inhibitor, rather than radiation therapy. Another patient’s posterior fossa tumor was found to have an *ATM* variant, confirmed on germline testing (patient 61, **Table 1**). If this variant had been identified prior to initiating therapy, the concern for radiation sensitivity with a germline *ATM* variant would have likely impacted management (9). Unfortunately, this result was not available prior to treatment, and the patient ultimately experienced radiation necrosis (10). Another patient with medulloblastoma was found to have a germline variant of uncertain significance (VUS) in *SUFU* (patient 83, **Table 1**). This prompted cascade testing in her mother who was found to have the same variant. The patient’s maternal grandmother had a basal cell carcinoma diagnosed in her 40s and reported several other clinical features that could be consistent with Gorlin syndrome, including an extra rib, extra teeth, and history of oophorectomy for ruptured ovarian cyst. For this reason, sequencing of her maternal grandmother’s basal cell carcinoma was performed, but the variant was not identified.

Twelve (39%) of the patients with somatic variants indicating a possible germline variant have not had confirmatory testing. Eight (26%) of these patients died before genetic counseling and germline testing coordination. Three (10%) of the patients had variants that were reclassified and ultimately did not have germline testing recommended. The remaining patient (3%) had insurance authorization for testing, which has not yet been performed. No patients who were offered confirmatory testing actively declined.

## Discussion

Molecular profiling of pediatric brain tumors is typically pursued with the priority of identifying potentially clinically significant variants. However, somatic sequencing may uncover potential germline variants, as demonstrated by this cohort. These variants can be important for informing a patient’s own cancer risk, can translate into life-saving surveillance and risk reduction interventions for self and family members, and – if identified in time – can sometimes impact therapeutic decisions (3, 4). The cohort on which we report includes several patients whose secondary germline findings could have influenced their treatment plan or surveillance care. Additionally, the majority of the patients with confirmed germline variants had at least one family member referred for genetic counseling and consideration of cascade testing. In one case, resultant cascade testing in family members assisted in diagnostic clarification (patient 83, **Table 1**). This patient, diagnosed with a medulloblastoma, was found to have a germline VUS in *SUFU.* Given the patient’s family history of basal cell carcinoma, one might suspect this variant was pathogenic. However, the variant was not detected upon subsequent testing of the grandmother’s basal cell carcinoma. Considering its absence in the grandparent’s tumor, along with the identification of a somatic *RELA* fusion in the patient’s recurrence, it was determined to be unlikely that the detected *SUFU* variant played a significant role in the development of this patient’s tumor.

Subsequent tracking of our cohort of patients highlights several challenges that arise with standard incorporation of tumor molecular sequencing into clinical care. In this protocol, the identification of somatic variants warranting germline follow-up testing was determined by the multi-disciplinary tumor board, a determination that might have variability across laboratories. Although the VAF was not used to exclude any variants in our cohort, it was documented for each case. Variants with an allele frequency of approximately 50% are generally assumed to be of germline origin; however, this threshold varies widely in the literature, ranging from 25-70% (8, 11–13) and is not the sole reliable indicator of need for germline testing (8). Tissue or tumor heterogeneity can result in a lower VAF (as can large indels, due to reference allele bias), and in some cases, copy number alterations, such as deletions, can mask germline point variants. It is therefore possible that potential germline variants are missed by laboratories using VAF thresholds to infer germline status or flag variants for germline follow-up. In our cohort, three of the confirmed germline variants (in two patients) had a corresponding tumor VAF less than 40%. One patient with a posterior fossa tumor treated with radiation therapy was found to have a variant in *ATM* with VAF of 25% (Patient 61, **Table 1**) (10). Had our standard process of evaluation used VAF alone, this patient would not have met the threshold for germline testing. Unfortunately, given delays in testing this variant was not discovered until after the patient had already received radiation treatment. The patient ultimately developed radiation necrosis – a long term effect noted more frequently in patients with germline *ATM* variants (9).

Given the challenges of using a somatic sample to identify which patients should undergo germline sequencing – including delays to confirmation of potentially time-sensitive, clinically relevant germline variants – some experts have advocated for upfront paired testing of both tumor and germline samples (14, 15). Paired testing is not without additional challenges. It is worth noting some laboratories intend their test to be only somatic and therefore intentionally restrict reporting likely constitutional variants, which can occur both in paired testing approaches and with bioinformatic filtering of potential germline variants (12). Aside from the technical and laboratory requirements/barriers, it necessitates a more nuanced informed consent process, which is already complicated by the distress of a new diagnosis or relapse of cancer (16, 17). ASCO guidelines recommend that oncology providers communicate the potential for incidental and secondary germline information to patients before they conduct somatic molecular profiling (4, 18). Further investigation is needed to establish more nuanced communication recommendations for oncologists to discuss the potential impacts of molecular sequencing testing and results with their patients and their families. Genetic counselors (GCs) have expertise in this communication, though their involvement may be limited early in the process by the urgency of this testing or at the time of return-of-results by GC availability, particularly at less resourced institutions. At our institution, this sequencing protocol led to institutional support for an integrated GC in our multidisciplinary neuro-oncology clinic. Incorporation of GCs into a multidisciplinary clinic in similar settings, such as adult gynecologic oncology clinic, improves adherence to genetic testing recommendations (19, 20). This and other quality improvement efforts in pediatric oncology clinics can be effective tools to improve uptake of genetic testing recommendations in pediatric patients with increased risk of cancer predisposition syndrome (21).

One identified barrier to patients undergoing appropriate follow-up testing in this cohort was patient death. Difficulty with follow-up testing when performed on a research protocol is further challenged when funding for administrative support does not continue following the completion of the research protocol. One patient who had prior insurance prior authorization for confirmatory germline testing but has not yet undergone the testing did not have a barrier identified in the medical record. It is possible that this might represent the family passively declining the recommendation. It is striking that all other patients who were recommended germline testing ultimately had a germline test performed. This might be related to the timing of this information in relation to their child’s initial diagnosis, with some not receiving the sequencing results and recommendation until after their child’s completion of upfront tumor-directed treatment. While for some families a delay in this discussion may have allowed more “mental space” to consider germline testing, others may have preferred to hear about this information sooner, particularly if the findings of the germline test had the potential to impact their child’s treatment plan. For those patients with confirmed germline variants, uptake of cascade testing in family members was variable. This is not unique to our cohort; in general, cascade testing remains suboptimal due to a variety of barriers at the patient-, provider-, and health systems-level (22–24).

The implications of this study should be considered in the context of a few limitations. First, this study reports on a small cohort of patients with brain tumors at a single institution. A limitation is that we did not consider germline testing of all variants found on somatic sequencing and therefore cannot validate our selection method prior to pursuing testing. Finally, the follow-up details of patient testing were determined by chart review, which may have simplified or misrepresented the extent to which the sequencing impacted the patients’ care or discussions about further testing.

Experience from this cohort highlights important considerations as upfront molecular sequencing becomes ever more frequent in childhood cancer care, both on clinical research protocols, such as the Molecular Characterization Initiative funded by the National Cancer Institute, and as part of the standard clinical care of patients (25). Additional research is needed to better delineate the impact of molecular sequencing both on patients’ clinical care and outcomes, as well as the psychosocial and financial impact on patients and their families.

## Data availability statement

The data analyzed in this study is subject to the following licenses/restrictions: available upon request. Requests to access these datasets should be directed to BG, brittany.greene@seattlechildrens.org.

## Ethics statement

The studies involving humans were approved by Seattle Children’s Hospital Institutional Review Board No. 00000506. The studies were conducted in accordance with the local legislation and institutional requirements. Written informed consent for participation in this study was provided by the participants’ legal guardians/next of kin.

## Author contributions

BG: Conceptualization, Data curation, Formal analysis, Visualization, Writing – original draft, Writing – review & editing. SS: Conceptualization, Data curation, Writing – original draft, Writing – review & editing. MT: Validation, Writing – review & editing. NW: Conceptualization, Writing – review & editing. BC: Data curation, Writing – review & editing. CL: Data curation, Writing – review & editing. VP: Data curation, Writing – review & editing. JB: Data curation, Writing – review & editing. AL: Writing – review & editing. JO: Writing – review & editing. RE: Writing – review & editing. JS: Data curation, Writing – review & editing. SL: Supervision, Writing – review & editing.
